# The Prevalence and Clinical Features of Skin Irritation Caused by Infection Prevention Measures During COVID-19 in Riyadh Region, Saudi Arabia

**DOI:** 10.7759/cureus.39846

**Published:** 2023-06-01

**Authors:** Muhannd M Alwadany, Manar A Alotaibi, Abdulelah S Almousa, Ahmed M Alawdah, Norah M AlDera, Ghadeer M Alqahtan, Heba Y Al Ajeel

**Affiliations:** 1 College of Medicine, King Faisal University, Alhofuf, SAU; 2 Department of Dermatology, Prince Sultan Military Medical City, Riyadh, SAU; 3 College of Medicine, Imam Mohammad Ibn Saud Islamic University, Riyadh, SAU; 4 College of Medicine, King Khalid University, Abha, SAU; 5 Dermatology, King Faisal University, Alhofuf, SAU

**Keywords:** saudi arabia, prevention, irritation, eczema, skin

## Abstract

Introduction: Coronavirus disease 2019 (COVID-19) or severe acute respiratory syndrome coronavirus 2 (SARS-CoV-2), caused by a novel coronavirus (CoV), was reported at the end of 2019 and caused a severe public health concern. It caused high mortalities by respiratory failure among infected people and was declared a pandemic by the World Health Organization (WHO) in March 2020. This virus caused infections through air or direct contact which documented a huge number of fatalities.

Aim: This study aims to examine the impact of the COVID-19 pandemic on skin eczema of the general public in the Riyadh region of Saudi Arabia.

Methods: This is a descriptive, cross-sectional, survey-based study, that was conducted via an online survey distributed to the general population of Riyadh for the period between January and February 2023. Data was collected through a questionnaire which was distributed through the social media websites.

Results: A total of 697 participants were involved in this study. Around one-fifth of the study participants (19.5%) reported that they suffer from some form of allergy and have family history of allergy (21.8%). Eczema was the most common type of allergy among the study participants accounting for 32.4%. A total of 116 participants (16.6%) reported that they have personal history of hand eczema or other skin disease on the hands. Cleaning and sterilization materials were reported as the most common cause of dryness and irritation of eczema (62.1%). Around 41.0% of the participants reported that they noticed a worsening of their symptoms after the pandemic, of which dryness was the most commonly reported sign noticed getting worse by 68.1% of the participants. The vast majority of the participants (89.7%) reported that new skin symptoms emerged on their hands after the beginning of the pandemic, of which dryness was reported by all the participants.

Conclusion: A considerable proportion of participants, particularly those with a history of hand eczema, experienced dermatological difficulties, including skin damage, due to the usage of COVID-19 preventive strategies. Thus, we recommend increasing the use of innovative infection prevention approaches and skin protection measures, such as regular hand hydration and maybe the use of less toxic skin disinfectants.

## Introduction

Severe acute respiratory syndrome coronavirus 2 (SARS-CoV-2), or coronavirus disease 2019 (COVID-19), was identified towards the end of 2019, causing a major public health risk [1]. The World Health Organization (WHO) declared a pandemic in March 2020 because of the significant mortality rates caused by respiratory failure among afflicted individuals [1]. This virus produced infections through airborne or direct contact, resulting in a staggering number of deaths [2]. WHO recommends using hand sanitizer, face masks, and social distance in order to reduce infections and remain safe [2]. Owing to the panic scenario, individuals employed regular hand washing (with alcohol and soap), face masks, and social isolation, which may have had adverse effects on their health, such as dermatitis or an allergy. According to one study, frequent hand sanitizer usage might lead to skin diseases [3]. It has been noted that the prevalence of dermatitis-sensitive individuals has increased. The health professionals who are already in more touch with patients and COVID-19 onset have been overloaded by this condition. Inflammation of the skin, such as eczema, was more prevalent in the general population and among healthcare professionals [3-5]. The skin barrier is the primary line of protection against antigens for the human body. Continuous hand-washing with chemicals or detergents, however, causes the epidermis to become brittle or sensitive, resulting in the progression of various illnesses or skin inflammations [6-8]. During the period of COVID-19, one study in China showed a 74.5% incidence of eczema among healthcare professionals [3]. In the western area of Saudi Arabia, one study by ZahrAllayali et al. (2021) indicated that 15.7% of participants with new instances of hand eczema and 34.5% of those with a history of hand eczema had their inflammation exacerbated [9]. Thus, it is advisable to take preventative steps, such as using skin moisturizers and limiting the usage of hand wash until absolutely required, to mitigate the negative effects of these hand sanitizers. Shelleh et al. (2004) observed a higher prevalence of dermatitis (34%) than other illnesses in the Najran General Hospital, Saudi Arabia, between 2000 and 2001 [10]. 

This study aims to examine the impact of the COVID-19 pandemic on skin eczema of the general public in the Riyadh region of Saudi Arabia.

## Materials and methods

This is a descriptive, cross-sectional, survey-based study, that was conducted via an online survey distributed to the general population of Riyadh for the period between January and February 2023. The study population consisted of all patients who live in Riyadh and met the inclusion criteria and were willing to participate in the study. The total number of Riyadh region is 10,500,000. The sample size calculation with a 99% confidence interval and 0.05 precision rate is 666.

Inclusion criteria were adults living in Riyadh city who are 18 years old and above, and exclusion criteria were any person who is under 18 or not willing to participate in the study.

Data was collected through a questionnaire which was distributed through the social media websites. Data were analysed using descriptive statistics. Categorical variables were presented as frequency and percentage.

The patients' confidentiality and the privacy of their data are the priority. Nothing leading to any ethical issue will be used, such as the names of participants. Ethical clearance was given by the ethical committee of the medical college at King Faisal University.

## Results

Table 1 below presents participants' demographic characteristics. A total of 697 participants were involved in this study. The majority of them (86.1%) were females and males were 13.9%. Almost 62.0% of them were aged 30 years and below. The majority of them (74.9%) were single and Saudi (74.3%) and non-Saudi was 25.7%. Only 5.7% of the participants reported that they are smokers. More than half of them (64.0%) were students. Around 4.2% of the participants were working in the healthcare sector, of which 62.1% were medical team members. Almost half (55.2%) of the workers in the healthcare sector were physicians. More than half (55.2%) of the healthcare workers reported that they use gloves for more than two hours on a daily basis. Almost one-third (34.5%) of the study participants reported that they wash their hands more than 20 times per day. Around 52.0% of them reported that they use hand sanitizer more than 10 times per day.

**Table 1:** Participants' demographic characteristics.

Variable	Frequency	Percentage
Gender		
Females	600	86.1%
Male	97	13.9%
Age groups		
18-20 years	223	32.0%
21-30 years	224	32.1%
31-40 years	83	11.9%
41-50 years	48	6.9%
51 years and older	119	17.1%
Marital status		
Single	522	74.9%
Married	160	23.0%
Widowed or divorced	15	2.2%
Nationality		
Saudi	518	74.3%
Non saudi	179	25.7%
Smoking status		
Smokers	40	5.7%
Profession		
Student	446	64.0%
Unemployed	85	12.2%
Education sector	70	10.0%
Administrative	67	9.6%
Healthcare professional	29	4.2%
For those who work in the healthcare sector		
Healthcare field (n= 29)		
Medical	18	62.1%
Surgical	7	24.1%
Laboratory	3	10.3%
Anaesthesia	1	3.4%
Specialty (n= 29)		
Physician	16	55.2%
Nurse	5	17.2%
Pharmacist	3	10.3%
Lab technician	2	6.9%
Dietitian	1	3.4%
Reception supervisor	1	3.4%
Radiologist	1	3.4%
Daily use of gloves )hours( (n= 29)		
Less than two hours	13	44.8%
2-5 hours	7	24.1%
More than five hours	9	31.0%
Number of times to wash hands per day: (n= 29)		
Less than 20 times	19	65.5%
More than 20 times	10	34.5%
The number of times you use hand sanitizer per day: (n= 29)		
Less than 10 times	14	48.3%
More than 10 times	15	51.7%

Allergy history of the study participants

Table 2 below presents the allergy history of the study participants. More than half of the study participants (54.7%) reported that they leave their house one to two times per week. Around one-fifth of the study participants (19.5%) reported that they suffer from some form of allergy and have a family history of allergy (21.8%). Allergic contact dermatitis was the most common type of allergy among the study participants accounting for 32.4%. Similarly, eczema was the common type of allergy as a family history among the study participants accounting for 57.9%.

**Table 2:** Allergy history of the study participants

Variable	Frequency	Percentage
How often do you leave the house during the week?		
1-2 times	381	54.7%
3-6 times	229	32.9%
More than 6 times	87	12.5%
Do you suffer from any kind of allergy?		
Yes	136	19.5%
What type of allergy do you have? (more than one answer could be chosen) (n= 136)		
allergic contact dermatitis	44	32.4%
Allergic rhinitis	32	30.1%
Asthma	41	23.5%
Others	43	31.6%
Do you have a family history with any kind of allergy?		
Yes	152	21.8%
What type of allergy do you have family history for? (more than one answer could be chosen) (n= 152)		
Eczema	88	57.9%
Asthma	81	53.3%
Allergic rhinitis	40	26.3%
Others	27	17.8%

Eczema history of the study participants

Table 3 below presents the eczema history of the study participants. A total of 116 participants (16.6%) reported that they have personal history of hand eczema or other skin disease on the hands. The vast majority of them (84.4%) reported that they use hand moisturizer. Around 78.4% of them reported that their eczema was diagnosed by a dermatologist and 66.4% of them reported that they follow up with a dermatologist. Most of the participants (80.2%) reported that their treatment was prescribed by a dermatologist. Only half of them (48.4%) reported that they are using the treatment prescribed by their dermatologist. One-third of them (33.6%) reported that they noticed an improvement in their eczema when they are away from work.

**Table 3:** Eczema history of the study participants.

Do you have a personal history of hand eczema or any other skin disease on the hands?		
Yes	116	16.6%
Do you use hand moisturiser?		
Yes	588	84.4%
Was the eczema diagnosed by a dermatologist? (n= 116)		
Yes	91	78.4%
Did you follow up with a dermatologist? (n= 116)		
Yes	77	66.4%
Was the treatment prescribed by a dermatologist? (n= 116)		
Yes	93	80.2%
Are you using the treatment prescribed by your dermatologist? (n= 93)		
Yes,	45	48.4%
Have you noticed an improvement in your eczema when you are away from work? (n= 116)		
Yes	39	33.6%
What are the causes of dryness and irritation of your eczema? (more than one answer could be chosen) (n= 116)		
Cleaning and sterilization materials	72	62.1%
Soap	50	43.1%
Water	39	33.6%
Certain foods, oils or herbs	33	28.4%
Animals	16	13.8%
Have you noticed a worsening of your symptoms after the pandemic? (n= 116)		
Yes	47	40.5%
If yes, what symptoms or signs did you notice getting worse? (more than one answer could be chosen) (n= 47)		
Dryness	32	68.1%
Severe itching	31	66.0%
Skin irritation and redness	29	61.7%
The appearance of rough patches or scales on the skin	27	57.4%
Pain or discomfort when touching the affected area	15	31.9%
Burning or pain	9	19.1%
Please choose the skin symptoms that match your skin damage: (n= 116)		
Redness	81	69.8%
Scales on the skin	63	54.3%
Cracked skin	61	52.6%
Open sores or infections	37	31.9%
Prominent bumps	34	29.3%
After the beginning of the pandemic, did you notice the emergence of new skin symptoms in the hands?		
Yes	104	89.7%
If yes, what symptoms or signs did you notice? (more than one answer could be chosen) (n= 104)		
Dryness	104	100.0%
Skin irritation and redness	63	60.6%
Severe itching	62	59.6%
The appearance of rough patches or scales on the skin	57	54.8%
Pain or discomfort when touching the affected area	23	22.1%
Burning or pain	22	21.2%
Please choose the skin symptoms that match your skin damage after the pandemic: (more than one answer could be chosen) (n= 116)		
Redness	87	75.0%
Cracked skin	63	54.3%
Scales on the skin	51	44.0%
Prominent bumps	32	27.6%
Open sores or infections	25	21.6%

Cleaning and sterilization materials were reported as the most common cause of dryness and irritation of eczema by the study participants accounting for 62.1%. Around 41.0% of the participants reported that they noticed a worsening of their symptoms after the pandemic, of which dryness was the most commonly reported sign noticed getting worse by 68.1% of the participants. The vast majority of the participants (89.7%) reported that new skin symptoms emerged on their hands after the beginning of the pandemic, of which dryness was reported by all the participants. Redness was the most commonly reported (75.0%) skin symptom that match the damage of the participants’ skin after the pandemic. 

## Discussion

Current recommendations from the WHO include social distancing, avoiding touching the face, wearing masks, wearing disposable gloves, and often washing hands with soap or alcohol-based disinfectants [11]. Although these treatments are suggested for preventing the spread of COVID-19, they may also have harmful effects on the skin. Following the WHO's recommendations, frequent handwashing (using soap and water for 40-60 seconds) has become the most important activity during the COVID-19 pandemic [11]; however, these measures may disturb the normal skin flora and natural protective skin barrier, leading to a variety of skin diseases, most frequently irritant and allergic contact dermatitis [3]. Globally, a prior study conducted in China found a 74.5% frequency of hand eczema among healthcare professionals during the COVID-19 pandemic, suggesting that the COVID-19 pandemic may be linked to skin problems [7].

In our study, more than half (55.2%) of the healthcare workers reported that they use gloves for more than two hours on a daily basis. Almost one-third (34.5%) of the study participants reported that they wash their hands more than 20 times per day. Around 52.0% of them reported that they use hand sanitizer more than 10 times per day. This was confirming the findings of previous studies which reported that participants tended to wash their hands more than 10-20 times per day during the pandemic [3,9]. The American Contact Dermatitis Society anticipates an increase in the incidence of both irritant and allergic contact hand dermatitis as healthcare workers and the general public concentrate on stringent hand hygiene. The Centers for Disease Control and Prevention advise routine handwashing with soap and water to prevent virus transmission [12]. In addition, long-term use of rubber gloves, especially without the application of an underlying moisturizer, worsens and/or raises the risk of allergic and irritating contact dermatitis [12].

In our study, allergic contact dermatitis was the most common type of allergy among the study participants accounting for 32.4%. The use of moisturizers has been advised to avoid hand eczema because high anxiety levels, excessive or irrational protective actions, and specific COVID-19 measures may cause or worsen the appearance of different dermatological diseases [12,13]. A prevalent condition that is considered to be of public health significance is hand eczema. Handwashing has been by far the most important risk factor for the occurrence of hand eczema. Frequent hand hygiene is another risk factor for hand eczema [13].

In our study, cleaning and sterilization materials were reported as the most common cause of dryness and irritation of eczema by the study participants accounting for 62.1%. Around 41.0% of the participants reported that they noticed a worsening of their symptoms after the pandemic, of which dryness was the most commonly reported sign noticed getting worse by 68.1% of the participants. Redness was the most commonly (69.8%) reported skin symptom that match the skin damage of the study participants. The vast majority of the participants (89.7%) reported that new skin symptoms emerged on their hands after the beginning of the pandemic, of which dryness was reported by all the participants. Redness was the most commonly reported (75.0%) skin symptom that match the damage of the participants’ skin after the pandemic. This was confirming the findings of a previous study in Saudi Arabia by ZahrAllayali et al. in 2021 [9]. In this study, the researchers observed that during the COVID-19 pandemic, 86 (13.1%) individuals had new-onset skin irritation, while 81 (31.3%) participants experienced a worsening of previously diagnosed hand allergic dermatitis. The most prevalent symptoms were dryness and a sense of constriction. Statistically, the frequency of handwashing rose after the commencement of the pandemic (p=0.001), and there was a correlation between new-onset symptoms and more frequent handwashing and the use of hand disinfectants during the pandemic (p=0.001) [9]. In previous studies, it was shown that itching, dryness, and soreness were the most typical clinical signs of skin injury among first-line healthcare providers [9,14].

This study is among the first few studies in Saudi Arabia that examined the prevalence and clinical features of skin irritation caused by infection prevention measures during COVID-19 in Saudi Arabia. At the same time, this study has limitations. A self-administered questionnaire through an online platform could be biased depending on the access to the internet among the study participants, and it may result in demographic bias by recruiting a younger, more internet-active population. Our study was cross-sectional, and participants were asked to complete an online questionnaire. As a result, a smaller percentage of people over 30 were included in the study. The two groups were not equally split by sex; more than 85% of participants were female. Consequently, the external generalizability may be impacted by both age and sex.

## Conclusions

Our research showed how COVID-19 prevention measures affected the skin of the general population. Due to the use of COVID-19 prevention measures, a significant portion of the participants especially those who had a history of hand eczema-reported dermatological issues, including skin damage. As a result, we advise promoting the use of novel infection prevention techniques and skin protection strategies, such as frequent hand hydration and maybe the use of less harmful skin disinfectants.
